# Who Can Get More Happiness? Effects of Different Self-Construction and Experiential Purchase Tendency on Happiness

**DOI:** 10.3389/fpsyg.2021.799164

**Published:** 2022-03-23

**Authors:** Aili Xie, Lianhua Liu, Shiqi Lyu, Lijuan Wu, Wen Tsao Pan

**Affiliations:** ^1^Guangzhou Huashang College, Guangzhou, China; ^2^Huashang Business Economic and Social Research Institute, Guangzhou, China; ^3^Guangdong University of Finance and Economics, Guangzhou, China; ^4^Guangdong University of Foreign Studies, Guangzhou, China

**Keywords:** self-construction, experiential purchase tendency, consumption effect, evaluation, experiential purchase propensity

## Abstract

This study introduces the self-construction methods of consumers and the tendency characteristics of experiential purchase to study the effects of physical purchase and experiential purchase on wellbeing. The dependent self-builders obtain higher happiness from experiential purchase; however, the independent self-builders get higher happiness from physical purchase. Furthermore, consumers with a high purchase experience get higher happiness from experiential purchase. Consumers with high material consumption tendency get significantly higher happiness than physical purchase from experiential purchase. Consumers with high materialism tendency gain higher happiness in experiential purchase, which is in line with the expectations of self-construction and consumption theories. This study provides the first evidence for the impact of self-construction methods on wellbeing with different consumption choices.

## Introduction

The disposable income of Chinese residents has been increasing since the mid-2010s. After meeting the basic living needs, they can consume more and more products to meet their material and spiritual needs. At this point, obtaining more happiness through consumption has become a research hot spot of scholars (Van Boven and Gilovich, 2003; Carter and Gilovich, 2012; Tully et al., 2015; Weidman and Dunn, 2016; Aknin et al., 2020). Current consumer psychology researchers mainly study the antecedents of consumption and the decision-making process (Howell and Hill, 2009; Dunn et al., 2011; Carter and Gilovich, 2014). The study has found that consumer personality traits can affect the wellbeing evaluation of consumer behavior, among which materialism is the most concerned trait of consumer personality traits, and materialism tends to buy in kind and obtain happiness by highlighting material possession (Van Boven and Gilovich, 2003; Tully et al., 2015; Weidman and Dunn, 2016).

Self-construction as an important personality trait of consumer self-mediation also has important implications for the wellbeing evaluation of consumer behavior. Research of scholars on how self-construction affects consumption patterns is mainly based on group research and individual consumer research. Based on the perspective of group study, Guo et al. (2010) studied the influence of self-construction on happiness by comparing the Chinese and the Western societies. Research on individual consumers is still relatively lacking. As a personality trait, experiential purchase tendency was proposed by Howell et al. (2012), and it also affects consumption tendency and consumption effect evaluation.

At this stage, due to the lack of research on the self-construction mode of consumers on consumption effect, this study explores the influence of self-construction mode and experiential purchase intention on consumer purchase intention and the evaluation of consumption effect through empirical research.

The rest of this study is organized as follows. The “Theoretical review and hypotheses” section reviews the theoretical background and explains the hypotheses in this study. The “Empirical results” section discusses the empirical results. The “Conclusion” section concludes.

## Theoretical Review and Hypotheses

### Happiness

Happiness is a very subjective positive experience after individuals evaluate their own life experiences according to their standards. According to the study by Diener (2000), individuals believe that their existing life is an affirmative attitude and feeling of their ideal life, where positive psychology calls subjective wellbeing (SWB).

Kahneman and Deaton (2010) suggested that happiness refers to the emotional state of a certain moment, called objective wellbeing (objective happiness), which is affected by situational behaviors such as certain display frameworks, i.e., prosocial behavior (Mogilner, 2010). The objective happiness measure requires the subject to score the current happiness, such as “Now, the degree of happiness you feel is?” The current happiness of subjects is measured by a 7-point Likert scale.

Happiness is a narrow concept than subjective happiness. There are many factors affecting happiness, and the most mentioned are material conditions and consumption, as well as family and social relations. Happiness increases with income and also decreases with income, and the relationship between income and happiness is shown in parallel in individuals with high-income levels. It is found that the overall national level of national happiness will not change over time and is not related to the increase of gross domestic product (GDP) *per capita*, which is called the “Easterlin Paradox.” Happiness comes from a balance of positive and negative emotions; although the concept of happiness is narrower than SWB, Aristotle equated happiness with subjective happiness. It is believed that subjective happiness is in a happy state, and despite the differences, the researchers have always taken “happiness” as a measure of happiness. Happiness tests characterize the wellbeing of people who are reporting the happiness they feel.

The concept of wellbeing adopts the concept of objective wellbeing, drawing on the wellbeing measurement scale developed by Kahneman and Deaton (2010), to better adapt to the experimental needs, “the degree of happiness you feel is? “Change to” the happiness you feel?” The current happiness of subjects is measured by a 7-point Likert scale.

### Experiential Purchase and Physical Purchase

Different consumers have different emphases on daily consumption tendencies. Some consumers tend to buy physical products such as computers, cars, and clothing. Some consumers tend to buy experience products such as tourism and outdoor activities. Scholars summarize this behavior phenomenon into two types of consumption, namely, experiential purchase, and physical purchase. Experiential purchase refers to purchases for life experience or experience, such as watching movies, traveling, and listening to concerts. Physical purchase refers to purchasing to own something, such as a bag and a mobile phone (Van Boven and Gilovich, 2003).

Different consumption tendencies bring different happiness. Physical purchase emphasizes the preservation and possession of goods. Also, in experiential purchase, consumers can only feel and enjoy in a limited time (Carter and Gilovich, 2012; Li et al., 2021). Different consumption tendencies have great differences in consumer happiness. Many studies have shown that experiential purchase brings more happiness than physical purchase. Scholars study the influence of different consumption activities on happiness from the perspective of self-determination theory, social comparison theory, and explanatory level theory (Dunn et al., 2011; Pham, 2015; O’Brien and Kassirer, 2019; Falk and Graeber, 2020; O’Neill, 2020; Ding et al., 2021; FitzRoy and Nolan, 2021). Personality traits are also an important factor in studying the impact of consumption patterns on happiness (Gilbert et al., 1995; Liberman and Trope, 1998; Deci and Ryan, 2000; Kumar and Gilovich, 2016; Mann and Gilovich, 2016; Matz et al., 2016).

Researchers study the impact of purchase types on the happiness of consumers from two large levels. Experiential purchase over time has different impact mechanisms from a physical purchase on happiness from the individual level. The positive experience is better over time, and even negative experiences will pass through time. Through the processing of the consumer, the memory becomes a special experience; experience is part of the self-concept of consumers, and the life of an individual is the synthesis of their life experience. Also, the physical purchase over time will gradually fade until the happiness of the consumer is lost. From the interpersonal perspective, experiential purchase is based on personal feelings, which is more subjective than social comparison. In contrast, the physical purchase is easy to reduce the happiness of consumers in the social comparison; experiential purchase is happier than physical purchase (Kumar and Gilovich, 2015, 2016), which puts forward the following assumptions according to the above theory in order to verify those assumptions through empirical research:

H1: Individuals get more happiness from experiential purchase than physical purchase.

### Interdependent Self-Construal and Independent Self-Construal

Markus and Kitayama (1991) put forward the concept of self-construction, based on cultural differences between China and the West on the formation of self-concept to represent the relationship between individuals and the outside world. Interdependent self-construal focuses on relationships with others and defines themselves through harmonious relationships (Markus and Kitayama, 1991). The authors usually represent themselves in interpersonal relationships and tend to experiential purchase. The experiential purchase is conducive to establishing social relations (Carter and Gilovich, 2014). Experiential purchase is also the process of establishing social relations.

Independent self suggests that the characteristics of every person are unique. When they respond to the social environment, the response is not only for the environment itself but also for the needs of the social situation which is also important. Still, they only serve as reference standards to strengthen the inner self. Independent self-builders can feel the immediate pleasure brought by consumption and are more likely to yield to the desire for consumption. The physical object has the characteristics of “here and now,” which is the objective existence of the present and can bring immediate pleasure to consumers.

Individuals define the self through their relationship with others, believing that neither self nor others can be separated from the situation but integrated with specific situations. The core concept is the harmony between self and others and creates harmonious relationships by adjusting themselves (Markus and Kitayama, 1991; Singelis, 1994). The individuals rely on others and adjust their behavior through their relationship with others and social situations. They attach importance to establishing relationships with others and various interpersonal relationship networks. The focus and core of the dependent self-builder is not the inner self but the collection of relationships between individuals and others. Lending to adjust themselves to create harmonious relationships, experiential purchase is conducive to build social relationships (Van Boven, 2005; Carter and Gilovich, 2014) experience consumption is easier to share, not only promote relationships, but also gain respect, and enhance individual happiness (Van Boven et al., 2010; Kumar and Gilovich, 2015).

Experiential consumption is easier for others to share, promoting relationships and gaining respect, conducive to improving individual wellbeing (Kumar and Gilovich, 2015). Independent self-construal is usually expressed themselves by internal traits. At the same time, physical consumption is a relatively isolated process, and the consumption process is also a process for consumers to establish self-awareness. Independent self-construal can feel the immediate pleasure brought by consumption and more likely to succumb to the desire for consumption. Physical objects have the characteristics of “here and now,” which is the objective existence of the moment and can bring instant pleasure to consumers. Based on this, the following assumptions are put forward:

H2: There are significant differences in the self-construction of happiness gained in different consumption activities.

H2a: Self-builders gain more happiness in their experience and buying.

H2b: Independent self-builders gain more happiness in physical purchases.

### Materialism and Experiential Purchase Tendency

Howell et al. (2012) first proposed an experiential purchase tendency. Individuals tend to buy experiential products in their daily purchases, paying more attention to their own internal experience than to their physical possessions. The experiential purchase tendency is also a personality trait. Different individuals are relatively stable and will not change easily (Howell and Hill, 2009). Unlike the repetition of purchase behavior of physical purchase, the experiential purchase of each tourism and each concert will bring great differences in different experiences for individuals. Many of these experiences are difficult to predict and cannot be repeated. They often give individuals more emotional stimulation and bring more happiness. High happiness leads to the more experiential purchase of individuals and forms a personality trait, which becomes a stable purchase tendency. The Experiential Buying Tendency Scale (EBTS) was developed by Howell et al. (2012).

Materialists (non-experience buyers) regard the acquisition of material wealth as the main goal of life struggle and even ignore other goals such as belonging and self-acceptance. Materialists have lower satisfaction with life and higher depression. They do not attach importance to their interpersonal relationship and are not satisfied with the quality of their interpersonal relationship (Kahneman and Deaton, 2010). Lack of trust in others, lack of social support, low sense of connection with others and society, lack of sense of belonging, and lack of social support lead to lower happiness. Although materialists believe that material possession can bring them the greatest happiness, when they are randomly assigned to physical purchase and experiential purchase groups, the subsequent happiness measurement shows that experiential purchase still brings them greater happiness (Carter and Gilovich, 2014). Materialism is not conducive to the improvement of the happiness of the people. Based on this, the following assumptions are made:

H3: The consumption tendency is different, and consumers feel different happiness in different ways of consumption.

H3a: Those who experience purchase tendency can get significantly more happiness in experiential purchase than physical purchase.

H3b: Materialists get significantly more happiness in experiential purchase than in physical purchase.

### Reliability and Validity Test

At present, the measurement of self-construal is mainly quantitative measurement. Singelis (1994) developed the self-construal scale (SCS) scale, which is widely used. The scale consists of 24 test items, of which 12 are independent self-construal and 12 are interdependent. This study uses the SCS scale. According to the self-constructed scale in Experiment 2, Cronbach’s alpha is 0.895, Kaiser-Meyer-Olkin (KMO) is 0.856, and *p* < 0.000, indicating that the reliability and validity of the scale are good, which is suitable for the analysis of this questionnaire.

According to the Richins’ Materialism Scale, which has nine items, the last is the reverse question, and Howell et al. (2012) developed the Experiential Purchase Tendency Scale (i.e., EBTS) that has four items. According to Materialism Scale and the Experiential Purchase Intention Scale in Experiment 3, Cronbach’s alpha of the Materialism Scale is 0.766, KMO is 0.836, and *p* < 0.000, and Cronbach’s alpha of the Experiential Purchase Tendency Scale is 0.812, KMO is 0.887, and *p* < 0.000, indicating that the reliability and validity of the two scales are good, which are suitable for the analysis of this questionnaire.

## Empirical Results

### Experiment 1

The purpose of this experiment is to verify hypothesis H1.

#### Pretest

The purpose of the pretest was to determine whether the description of “physical purchase” and “experiential purchase” in Experiment 1 was appropriate. This study used the method developed by Carter and Gilovich (2012) to investigate experiential and physical purchases. Let the subjects read, imagine you are making a purchase decision: You are going to buy a new phone.

Here are two descriptions. Description 1: the mobile phone you are about to buy is a relatively high-end mobile phone in the market. The metal fuselage, ceramic touch, fuselage fine grinding, corrosion resistance, high stability of the six-layered spraying process, and unique camera design make the vision clearer and wider. Everyone knows that the mobile phone is very advanced. When you hold it in your hand, it is very attractive.

Description 2: the mobile phone you are about to buy is a relatively new and high-end model. The screen of the mobile phone is very touchy and warm. The camera shooting function of the mobile phone is more powerful, and it can take a clearer picture of the wonderful moments in your life. It can also edit photos according to your preferences to have more different and better experiences.

Then, let the pretest subjects answer the following two questions: (1) you think description 1 focuses more on physical description or experience description, using a 7-point Likert scale (1 = physical description and 7 = experience description) score and (2) you think description 2 focuses more on physical description or experience description, using Likert scale score. Through the analysis of 40 sample data, M_physical_ = 2.55, which is significantly lower than the median value 4 (*t* = -8.453, *p* < 0.000), indicating that description 1 is the physical description in manipulation. M_experience_ = 5.88, significantly higher than the median 4 (*t* = 12.297, *p* < 0.000), indicates that description 2 is the experience description in manipulation, and the data show that the manipulation is successful, so in the experiment, description 1 is purchased as physical, and description 2 is purchased as experience.

### Experimental Participants and Procedures

The formal participants were 135 undergraduate students from a university in Guangzhou, with 119 valid respondents, including 43 male students (36.1%) and 76 female students (63.9%), with an average age of 21.46 years (*SD* = 3.46).

Step 1: Tell the subjects that we are experimenting on consumption choices. First, let all the subjects read the above materials, and then, let the subjects rate description 1 and description 2 belonging to physical description or experience description, respectively.

Step 2: Let the subjects choose one of their favorite descriptions from the above, using the scale developed by Carter and Gilovich (2014), and ask the subjects to score the degree of happiness they feel at present (e.g., “Now, what is the degree of happiness you feel?” 1 means very unhappy, and 7 means very happy).

Step 3: Finally, ask the subjects whether they know the purpose of the investigation, let them fill in the personal data.

### Experimental Result Analysis

#### Manipulation Test

Subjects were asked to score whether descriptions 1 and 2 belonged to physical or experience descriptions; description 1 belonged to physical or experience description, M_physical_ = 2.45, which was significantly lower than the median value 4 (*t* = 12.65, *p* < 0.000), whereas description 2 belonged to physical or experience description, M_experience_ = 5.45, which was significantly higher than the intermediate value 4 (*t* = 8.43, *p* < 0.000). The manipulation test data show that physical description and experience description are successfully manipulated.

#### Hypothesis Testing

By calculating the average happiness of the participants who choose physical purchase and those who choose experiential purchase in the questionnaire, the descriptive statistical scale in Table 1 is obtained as follows. One-way ANOVA showed that *F*(1, 117) = 54.25, *p* < 0.000, M_physical_ = 4.03, (*SD* = 1.38), and M_experience_ = 5.72, (*SD* = 1.04), indicating that there was a significant difference in happiness between physical purchase and experiential purchase. Thus, hypothesis H1 was verified.

**TABLE 1:** Descriptive statistics of the difference in happiness.

Consumption type	M	SD	N	95% Confidence interval	
				Lower limit	Upper limit
Physical purchase	4.027	0.191	62	3.649	4.405
Experiential purchase	5.720	0.128	57	5.466	5.973

The experiment verifies that “experiential purchase” can bring consumers more pleasure and happiness than physical purchase (Van Boven and Gilovich, 2003; Howell and Hill, 2009; Carter and Gilovich, 2012). Purchasing in-kind fades away with time, individual experience can become part of the individual concept through memory. The life experience of a person is their life, so the experiential purchase can bring an individual more happiness.

### Experiment 2

This experiment verifies the hypotheses H2, H2a, and H2b that self-construction of different people in the happiness of physical and experiential purchase significantly differs.

The formal participants were 245 undergraduate students from a university in Guangzhou, with 237 valid respondents, including 87 male students (36.7%) and 150 female students (63.3%), with an average age of 21.22 years (*SD* = 3.62).

#### Experimental Participants and Procedures

Step 1: Tell the subjects that we are experimenting on consumption choices to get the subjects to make a purchase decision. The introduction to the experiment is as follows: Imagine that you are facing a purchase challenge right now because budgets are limited, and you can only choose one. You are considering buying a branded mobile phone with a powerful CPU, memory, storage, the favorite imaging quality of an industry, IOS program response speed and memory management, and strong product hedging capabilities. At the same time, you also consider that if you do not change your new mobile phone, you can use this expenditure to travel to Tibet, where there are continuous snow mountains, quiet lakes, seemingly reachable clouds, azure sky, and the Potala Palace, which has long been your dream. Then, let the subjects choose, fill in the happiness scale developed by Kahneman and Deaton (2010), and fill in the Single SCS scale.

Step 2: Let the subjects score the degree of happiness that the decision can feel.

Step 3: Finally, ask subjects if they know the purpose of the investigation and fill out the personal information.

#### Experimental Data Analysis

This experiment adopted 2 (construction mode) × 2 (consumption type) between-group experiments. First, the reliability and validity of the self-construction scale in the questionnaire were analyzed. The Cronbach’s alpha was 0.895, and the KMO was 0.856 (*p* < 0.000), indicating that the reliability and validity of the scale were good, which was suitable for this survey.

Through simple descriptive analysis of the happiness of different consumption types under different construction types, Table 2 is obtained.

**TABLE 2:** Descriptive statistics of the difference in happiness.

Construction types	Consumption type	*M*	*SD*	N	95% Confidence interval	
					Lower limit	Upper limit
Dependency construction	Physical purchase	4.581	1.222	62	4.355	4.806
	Experiential purchase	5.625	0.669	96	5.444	5.806
Independent construction	Physical purchase	5.694	0.738	32	4.779	5.408
	Experiential purchase	4.723	0.615	47	4.464	4.983

**FIGURE 1:**
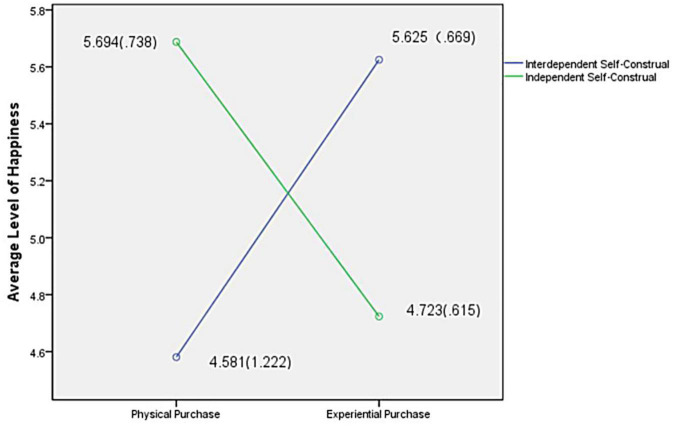
Interaction between constructive style and consumption type.

Taking the happiness degree of the subjects as the dependent variable and the construction type and the consumption type as the independent variables, the single-factor ANOVA is carried out. The results showed that the interaction between different construction methods and consumption types was significant *F*(1, 233) = 70.805, *p* < 0.000, as shown in Figure 1. The M_experience_ of the dependent construction is 5.625, and the M_physical_ is 4.581, whereas the M_experience_ of the independent construction is 4.723, and the M_physical_ is 5.694, *F*(1, 233) = 31.09, *p* < 0.000, which has a significant difference. Self-construal of different people in different consumption patterns of happiness has been significantly different, and hypothesis H2 is verified.

In the context of dependent construction, the happiness level of consumption patterns was significantly different *F*(1, 156) = 33.162, *p* < 0.000, indicating significant differences in the degree of happiness in the same self-construction context experienced by participants in different consumption types. The degree of happiness experienced by the physical purchase subjects M_physical_ = 4.58 (*SD* = 1.222), the degree of happiness experienced by the experimental purchase subjects M_experience_ = 5.63 (*SD* = 0.669), and the degree of happiness between experiential purchase and physical purchase is significant [*t*(95) = 15.308, *p* < 0.000]. This evidence shows that in the interdependently constructed group of subjects, the degree of happiness that the subjects feel from experiential purchases is significantly higher than that of physical purchases. It is assumed that the happiness of H2a-interdependent self-construal in the experiential purchase is significantly higher than that in physical purchase.

In the context of independent self-construal, there is a significant difference in the level of happiness among different consumption types *F*(1, 77) = 3.191, *p* < 0.000, indicating that in the context of independent self-construal, there is a significant difference in the degree of happiness that physical purchase and experiential purchase subjects can experience. The degree of happiness that M_physical object_ is 5.69 (*SD* = 0.738), the degree of happiness that M_experience_ is 4.72 (*SD* = 0.615), and the degree of happiness that physical purchase and experiential purchase are significantly different [*t*(31) = 7.418, *p* < 0.000]. The results showed that subjects felt more happiness from the physical purchase than physical purchase in the independent self-construal group. It is assumed that H2b-independent self-construal obtains significantly higher happiness in physical purchase than experiential purchase.

Experiment 2 explored the difference in happiness between consumers with different self-construction methods in the physical purchase and experiential purchase. The interdependent self-construal indicated more happiness in the experiential purchase, while the interdependent self-construal obtained more happiness in physical purchase. The interdependent self-construal tended to define themselves through the relationship with the outside world. The experience consumption process is social, increasing interpersonal intimacy to meet the interdependent self-construal needs for social relations and improve their happiness. Independent self-construal pays more attention to themselves, and the physical purchase is often completed alone, which can meet the needs of independent self-construal self-realization. Thus, the hypotheses H2, H2a, and H2b are verified.

### Experiment 3

The purpose of this experiment was to verify H3, H3a, and H3b that people with different materialistic tendencies and experiential purchase tendencies have significant differences in obtaining happiness in the physical purchase and experiential purchase.

#### Experimental Participants and Procedures

The participants in the formal experiment were 268 undergraduate students from a university in Guangzhou, 256 valid respondents, 116 male students (45.3%), and 140 female students (54.7%), with an average age of 21.66 years (SD = 4.646).

Step 1: Tell the subjects that we are experimenting on consumption choices and get them to make a purchase decision. The preamble to the experiment is as follows: Suppose you are facing a dilemma in consumption decisions because budgets are limited, and you can only choose one from two. Listen to the concert of a favorite singer or buy a large brand of clothing that you have long loved. The concert will make you happy. You can melt yourself into music and embark on a dream journey with melody. And you have a long-loved brand of clothing, and it has a unique design and superior supplies, which will make you feel that you have very grade clothes. Then, let the subjects choose whether they would like to go to the concert or buy their favorite clothes.

Step 2: Let subjects score the degree of happiness based on the above decisions.

Step 3: Then, let the subjects fill in the Materialism Scale and the Experiential Purchase Tendency Scale.

Step 4: Finally, ask the subjects whether they know the purpose of the investigation and fill out the personal information.

#### Experimental Data Analysis

This experiment adopted 2 (experiential purchase tendency, materialism consumption tendency) × 2 (consumption type) between-group experiments. First, the reliability and validity of the Materialism Scale and the Experiential Purchase Tendency Scale were analyzed. The Cronbach’s alpha of the Materialism Scale was 0.766, and the KMO was 0.836, *p* < 0.000. The Cronbach’s alpha of the Experiential Purchase Tendency Scale was 0.812, and the KMO was 0.887, *p* < 0.000, indicating that the reliability and validity of the two scales were good, which were suitable for the analysis of this questionnaire. Variables are calculated and converted for the reverse questions of the Materialism Scale. Then, a simple descriptive analysis is carried out on the happiness obtained by participants with different consumption tendencies and consumption types, as shown in Table 3.

**TABLE 3:** Descriptive statistics of the difference in happiness.

Propensity to consume	Consumption type	*M*	*SD*	N	95% Confidence interval	
					Lower limit	Upper limit
Experiential purchase tendency	Experiential purchase	6.174	0.677	46	5.841	6.507
	Physical purchase	5.423	1.027	26	4.980	5.867
Physical purchase tendency	Experiential purchase	5.962	1.123	104	5.740	6.183
	Physical purchase	5.413	1.402	80	5.160	5.665

**FIGURE 2:**
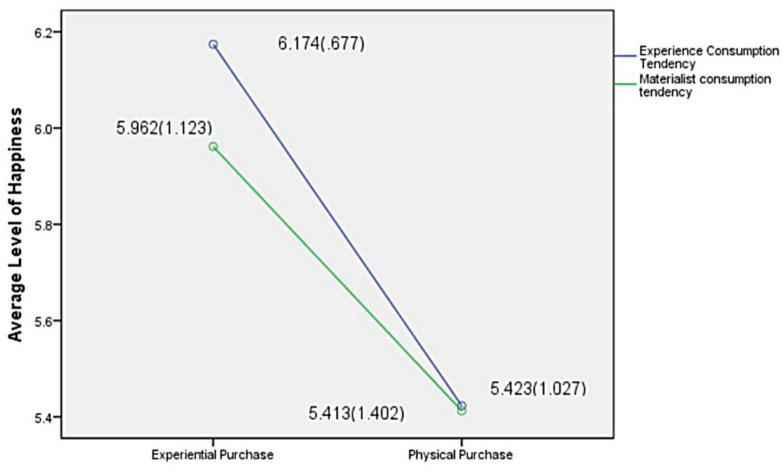
Interaction between consumption tendency and consumption type.

The results showed that the interaction between different consumption tendencies and consumption types was insignificant *F*(1, 252) = -1.763, *p* = 0.185, as shown in Figure 2. One-way ANOVA was conducted with the happiness of participants as the dependent variable and consumption propensity and consumption type as the independent variables. M_experience_ of experiential purchase propensity = 6.174, M_physical_ = 5.423, M_experience_ of materialism consumption propensity = 5.962, M_physical_ = 5.413, *F*(1, 252) = 15.527, *p* < 0.000, showing significant differences. Assuming that H3 has a different propensity to consume, the significant difference in happiness obtained by participants in the experiential purchase and physical purchase is verified, indicating that participants with different propensity to consume have a significant difference in happiness obtained in the experiential purchase and physical purchase.

In the context of experiential purchase tendency, the main effect of the level of happiness of consumption types was significant *F*(2, 70) = 10.226, *p* < 0.002, indicating that, in the context of high experiential purchase tendency, there were significant differences in the degree of happiness that different consumption types of subjects can experience. The degree of happiness experienced by physical purchase subjects was 5.42 (*SD* = 1.027), the degree of happiness experienced by experiential purchase subjects was 6.17 (*SD* = 0.677), and the difference between experiential purchase and the physical purchase was significant [*t*(46) = 7.556, *p* < 0.000]. This evidence shows that subjects with high experiential purchase tendency feel more happiness from the experiential purchase than physical purchase. Assuming that H3a is verified, experiential purchasers tend to enjoy experiential purchases significantly higher than physical purchases.

In the context of physical consumption tendency, the main effect of the happiness level of consumption types was significant *F*(2, 182) = 9.324, *p* < 0.003, indicating that in the context of high physical consumption tendency, there was a significant difference in the degree of happiness that physical purchase and experiential purchase subjects can experience. The happiness level of physical purchase subjects was 5.41 (*SD* = 1.402), the happiness level of experiential purchase subjects was 5.96 (*SD* = 1.123), and the happiness of different consumption types was significantly different [*t*(103) = 5.010, *p* < 0.000]. This result shows that subjects with a high propensity to physical consumption still feel significantly higher happiness from the experiential purchase than physical purchase. Assuming that H3b is verified, consumers with a high propensity to physical consumption get a higher happy experience from experiential purchase.

The experimental data show that subjects with different consumption tendencies have significant differences in happiness in physical and experiential purchases. Assuming that H3 is validated, subjects with a higher propensity to experiential purchase gain more happiness from experiential purchase. Assuming that H3a is verified, subjects with higher materialistic propensity to consume also have significant differences in happiness from experiential purchase and physical purchase, and experiential purchase brings happiness to subjects significantly higher than physical purchase. We observed that H3b is verified. Although participants with high materialistic consumption tendency expect to obtain more happiness from physical consumption, they often fall into the “materialistic supremacy” trap. Excessive pursuit of material satisfaction is self-defeating behavior, which brings more negative emotions and depression. Experiential purchase is a kind of social consumption, which can meet the social needs of materialism and correct their extreme emphasis on material pursuit and neglect of the construction of interpersonal relationships. Therefore, subjects with a high propensity to materialistic consumption have significantly higher pleasure in experiential purchase than physical purchase.

## Conclusion

The main conclusions are as follows: interdependent self-construal gains more happiness from experiential purchase, and independent self-construal gains more happiness from physical purchase. Subjects with a high propensity to experiential purchase gain more happiness from experiential purchase, and subjects with materialism tend to gain more happiness from experiential purchase.

First, it enriches the related research of physical purchase and experiential purchase. Previously, the research on the influence of physical purchase and experiential purchase on the happiness of consumers mainly analyzed the influence of experiential purchase and physical purchase on happiness from construal level theory, self-concept, social comparison, and self-determination theory. This study introduces the self-construction of the personality traits of consumers, and consumption tends to study and makes an empirical study on the influence of different construction methods on consumption types and the influence of different consumption tendencies on consumption types, which enriches the related research of physical purchase and experiential purchase.

Second, it reveals the influence of different construction methods on consumption types, which affects the explicit behavior and implicit psychology of the consumers. Experiment 2 proves that interdependent self-construal has a significantly higher degree of pleasure in experiential purchase than physical purchase because interdependent self-construal defines themselves by the relationship with others, they attach importance to the relationship with others, the core of their attention is to create a harmonious interpersonal relationship, and experiential purchase due to its social can meet the needs of interdependent self-construal of interpersonal relationships and can increase the intimacy between people. Independent self-construal is more likely to experience the immediate pleasure of physical purchases. Physical purchase is usually an individual behavior, which is relatively isolated. Independent self-construal perceives the difference between themselves and the outside world in the physical purchase and obtains more happiness.

Third, it enriches the research on materialism and consumption propensity. Researchers often suggest that materialism is a personality trait but ignore that experiential purchase propensity is also a personality trait that significantly affects the purchase behavior of consumers. This study verifies through experiments that although materialists attach importance to physical purchase, the physical purchase cannot bring the expected happiness, the experiential purchase can help materialists establish the relationships of family and friends, and the support of family and friends can promote the improvement of happiness.

It is also important to pay attention to consumer experience, the consumer experience as an important element of enterprise marketing strategy to manage, enterprise product form whether pure physical products, physical products with experience or experience products, consumers will produce use experience, consumer experience is good or bad affect the enterprise product sales. From the enterprise level, we should pay attention to consumer experience and have channels to collect and understand consumer product experience, pay attention to consumer experience feedback, and constantly improve the consumer experience. Pure physical products should increase the function or characteristics to stimulate consumer experience. Experience products should pay attention to the feelings brought to consumers in the experience process and constantly improve or upgrade experience to avoid the negative impact of repeated experience on consumers.

In addition, the personality traits of consumers are fully considered to incorporate advocacy and packaging strategies. Self-construal consumers tend to focus on the context of stimulus, and their motivation to consume is often concentrated on sharing and maintaining contact with others. They pay more attention to the social experience of products, so such consumer companies can strengthen the value of products to promote interpersonal relationships, i.e., presenting social scenes and slogans to stimulate audience awareness and recognition of products. The background rarely stimulates consumers with independent self-construal. They pay more attention to self-expression and possession of things. Their uniqueness should be highlighted in publicity and packaging for such consumer products and the significance of self-representation to consumers.

The experimental design method adopted in the study is to construct the consumption type through the literal description, which is not a real shopping scene. Participants only use literal reading to virtualize shopping choices. Therefore, different results may appear in the real consumption scene. Future research can be based on real consumption scenes and real consumption. As a personality trait, materialism has been paid much attention to and studied by the academic circle.

Finally, Howell et al. (2012) explored the psychological formation mechanism of consumers experiencing purchase intention from the reward mechanism, and more psychological mechanisms are worth studying. Nowadays, the lifestyle of “minimalism” is popular in many countries, abandoning the redundant material experience of the simple life. Consumers in many countries show the pursuit of material and fanaticism in the early stage of rapid development. With the continuation of wealth, the pursuit of material of consumers is gradually reduced, which shows that high materialism consumption tendency will transform to experiential purchase tendency. In contrast, as a personality trait, experiential purchase tendency has not been paid attention to; however, in reality, consumers of this type pay less attention to physical purchases and pay more attention to their inner self-feelings.

## Data Availability Statement

The original contributions presented in the study are included in the article/supplementary material, further inquiries can be directed to the corresponding author/s.

## Author Contributions

AX was responsible for the questionnaire design of the article. LL, SL, LW, and WP were responsible for the data collection of the article and the writing of the article. All authors contributed to the article and approved the submitted version.

## Conflict of Interest

The authors declare that the research was conducted in the absence of any commercial or financial relationships that could be construed as a potential conflict of interest.

## Publisher’s Note

All claims expressed in this article are solely those of the authors and do not necessarily represent those of their affiliated organizations, or those of the publisher, the editors and the reviewers. Any product that may be evaluated in this article, or claim that may be made by its manufacturer, is not guaranteed or endorsed by the publisher.
